# Prevalence and factors associated with overweight and obesity in selected health areas in a rural health district in Cameroon: a cross-sectional analysis

**DOI:** 10.1186/s12889-021-10403-w

**Published:** 2021-03-10

**Authors:** Larissa Pone Simo, Valirie Ndip Agbor, Francine Zeuga Temgoua, Leo Cedric Fosso Fozeu, Divine Tim Bonghaseh, Aimé Gilbert Noula Mbonda, Raymond Yurika, Winfred Dotse-Gborgbortsi, Dora Mbanya

**Affiliations:** 1 Clinical Research Education, Networking & Consultancy (CRENC), Douala, Cameroon; 2 Clinical Trial Service Unit and Epidemiological Studies Unit, Nuffield Department of Population Health, University of Oxford, Oxford, UK; 3 Department of Clinical Research, Health Education and Research Organization (HERO), Douala, Cameroon; 4 Heart and Life Foundation, Douala, Cameroon; 5 Bafmen Sub-divisional Hospital, Bafmen, Northwest Region Cameroon; 6 Department of Public Health, Faculty of Medicine and Biomedical Sciences, Yaoundé, Cameroon; 7 Technical Group for Tuberculosis Control, North West Regional Delegation of Public Health, Bamenda, Cameroon; 8 School of Geography and Environmental Science, Highfield, University of Southampton, Southampton, SO17 1BJ UK; 9 Faculty of Health Sciences, the University of Bamenda, Bamenda, Cameroon; 10 Yaoundé University Teaching Hospital (YUTH), Yaoundé, Cameroon

## Abstract

**Background:**

Overweight and obesity are major public health problems worldwide, with projections suggesting a proportional increase in the number of affected individuals in developing countries by the year 2030. Evidence-based preventive strategies are needed to reduce the burden of overweight and obesity in developing countries. We assessed the prevalence of, and factors associated with overweight and obesity in selected health areas in West Cameroon.

**Methods:**

Data were collected from a community-based cross-sectional study, involving the consecutive recruitment of participants aged 18 years or older. Overweight and obesity were defined according to the WHO classification. The statistical software R (version 3.5.1, The R Foundation for statistical computing, Vienna, Austria) was used for statistical analysis. Multivariable logistic regression analysis was used to assess independent factors associated with overweight and obesity, and obesity.

**Results:**

Records of 485 participants were included for analysis. The age and sex-standardized prevalence of overweight, obesity, and overweight and obesity were 31.1% (95% CI, 27.0–35.2), 18.9% (95% CI, 14.9–22.9), and 50.1% (95% CI, 45.7–54.6), respectively. In multivariable analysis, being female (adjusted OR [aOR] = 2.79, 95% CI = 1.69–4.63), married (aOR = 3.90, 95% CI = 2.23–6.95), and having secondary or tertiary education (aOR = 3.27, 95% CI = 1.77–6.17) were associated with higher odds of overweight and obesity, while current smokers had lower odds of overweight and obesity (aOR = 0.37, 95% CI = 0.16–0.82) when compared to their respective counterpart. Compared to their respective reference categories, being female being (aOR = 3.74, 95% CI = 2.01–7.30), married (aOR = 2.58, 95% CI = 1.37–5.05) and having secondary or tertiary education (aOR = 2.03, 95% CI = 1.00–4.23) were associated with higher odds of obesity after adjustments for confounding.

**Conclusion:**

We observed a high prevalence of overweight and obesity in this study. The odds of overweight and obesity was higher in females, married participants, and those with higher levels of education. Community-based interventions to control overweight and obesity should consider targeting these groups.

**Supplementary Information:**

The online version contains supplementary material available at 10.1186/s12889-021-10403-w.

## Background

Overweight and obesity result from excessive accumulation of fat in the body [1], and are associated with increased risk of non-communicable diseases such as cardiovascular diseases, diabetes, cancers and other health-related complications [2–4]. In 2005, 30.0% of the world’s adult population was overweight or obese, with this value estimated to almost double by 2030 [5]. The World Health Organization (WHO) estimated that about two billion and 600 million adults worldwide were overweight and obese in 2014, respectively [6]. Although overweight and obesity are more common in economically developed countries, developing countries have been projected to have a much larger proportional increase in the number of overweight and obese individuals between 2005 and 2030 [5].

Expanding westernization and urbanization in sub-Saharan Africa, which are associated with unhealthy dietary habits and sedentary lifestyle, are fuelling the prevalence of obesity in the Region [7, 8]. Abubakari et al in 2008 estimated the prevalence of obesity among West African adults at 10% with females being more likely to be obese than their male counterparts [9]. More recent data revealed the prevalence of obesity to be 17.8, 30, and 33.7% in some populations of Ghana, South Africa and Nigeria, respectively [10–12]. According to a meta-analysis by Ofori-Asenso et al 2016, almost half of Ghanaians were either obese or overweight [13]. Also, they reported a prevalence of obesity and overweight of 25.4 and 17.1%, respectively. Being female, living in urban areas, being married, higher socioeconomic status, higher educational status, and physical inactivity have been associated with increased odds of being overweight or obese [11–14].

Cameroon has not been spared by the wave of westernization and urbanization. According to the Food and Agricultural Organisation (FAO), the prevalence of obesity in the adult Cameroonian population increased steadily from 4.9% in the year 2000 to 9.5% in 2016 [15]. Fezeu et al reported a two-folds increase in the prevalence of age-standardized prevalence of overweight and obesity in rural Cameroon over a period of about 10 years [16]. These reports highlight the fact that the obesity epidemic is increasingly taking an important place as a cause of disease burden in Cameroon and may deteriorate if no action is taken. While this condition has been explored in several sub populations in Cameroon with a few in the general population [16–20], there is still a dearth of information on the topic in rural areas of the country. Furthermore, the national health development plan by Cameroon’s ministry of public health is yet to implement policies to address the rising prevalence of obesity in rural Cameroon [21].

Understanding the burden and determinants of obesity in the rural population is critical to guide the implementation of evidence-based health policies and community-based prevention and control strategies against obesity. This study sought to determine the prevalence of and factors associated with overweight and obesity in selected health areas in the Baham Health District in the West Region of Cameroon.

## Methods

### Study design and population

We conducted a community-based cross-sectional study between August and October 2018 in the Baham Health District (BHD), a rural area in the West Region of Cameroon [22]. This study was conducted as part of the University of Bamenda Medical Students Association (UBaMSA) annual community health campaign – which sorted to provide free screening for major preventable conditions such as hypertension, diabetes, and viral hepatitis. In addition, the campaign sought to offer medical advice and free medical and surgical care or linkage to appropriate care and long term follow-up. The Baham Health District has nine health areas and an estimated population of 51,500 in 2001 [22]. Baham is located about 250 km from Douala, the economic capital of Cameroon, and 20 km from Bafoussam, the regional capital of the West Region [22], Additional file 1. The West Region of Cameroon, and the Baham Health District, is inhabited by the Bamileke, which is a group comprised of many tribes [23]. The Bamileke group was formed by the amalgamation of natives from the West, North-west, and South-west regions of Cameroon [23]. French is the primary language of communication of the population, but a few speak English and the local Lingua Franca – Pidgin. Maize, cassava, and potatoes, which are carbohydrate-rich foods, are the staples, supplemented with beans and peanuts. Farming and trading of the products is the principal income-generating activity of the people. Participants were recruited from four of the nine health areas of the BHD, including the Bahiala Cheffou, Bapa, Baham, and Ngouogoua health areas. This study entailed a cross-sectional analysis of data collected for the primary study [24].

Ease of accessibility to the health areas was the criteria of selection of the health areas. For this study, we consecutively included participants at least 18 years of age with available data on weight and height sufficient to compute the body mass index.

### Study procedure and data collection

A month before the UBaMSA health campaign, community members were sensitized on the aims and the period through which campaign will run through mass communication (through the local radio stations) and interpersonal communication (using community social mobilizers). The community social mobilizers went around each quarter and to farms with a megaphone for three consecutive days to sensitize the public. They also targeted various meeting houses to sensitize the community-dwellers. It was emphasized that community member will not be charged for any screening test or treatment performed during the campaign. Participants were invited to state-owned health facilities, where the health team was based, of each health area for data collection. The health campaign lasted for 10 days.

Data collection was guided by the three-step WHO STEPwise approach to Surveillance (STEPS). Data were collected by trained medical students and doctors from the University of Bamenda, Cameroon, who were trained on the WHO STEPS approach to data collection by the principal investigator. In step one, we collected participants’ sociodemographic and clinical information through face-to-face interviews and with the use of a pre-designed questionnaire adapted from the WHO STEPwise approach. Recorded information included age (in years), sex, marital status, occupation, level of education, and family history of hypertension. Information on participants’ lifestyle such as smoking habits (previous smoker, current smoker [Yes/No], number of cigarettes smoked per day [for current smokers], duration of smoking in years), alcohol consumption (Yes/No), units of alcohol consumption, duration of alcohol consumption in years, vegetable and fruit consumption per week, and intensity of physical activity were also recorded. A translator was used in cases where participants did not understand either English or French. A signed or verbal informed consent was obtained from all participants prior to the interview.

In step two, anthropometric measurements were done using standard methods; a calibrated stadiometer was used to measure height to the nearest 0.1 cm. Weights were measured to the nearest 0.5 kg using an automated scale, and participants were allowed to mount the scale in light clothing.

### Definitions

1. Body mass index (BMI) was calculated with the following formula: *weight (kg)/ height (m)*^*2*^*.* BMI based body habitus (in kg/m^2^) was further categorized as underweight (BMI < 18.5), normal weight (BMI = 18.5–24.9), overweight (BMI = 25.0–29.9), and obese (BMI ≥30) [25].
2. Occupational level was categorized into “low” (no technical know-how or expert training required, e.g. manual workers), “medium” (requiring a degree of technical know-how but no expert training, like salesmen, and bike and taxi drivers) and “high” (major professionals requiring advanced training like teachers, health personnel, and accountants).
3. The intensity of physical activity was categorized into “moderate” (e.g. brisk walking, moderate farm work like weeding and harvesting, hunting, lifting masses < 20 kg, housework and domestic chores, and general building tasks such as roofing and painting) and “vigorous” (running, briskly ascending and descending hills, intense farm work such as manual tilling of the soil, digging ditches and carrying masses > 20 kg) [26]. “Low physical activity” was considered as a sedentary lifestyle at home and at work.
4. Alcohol consumption in units of alcohol consumed per week was computed: *5% x volume of beer (in ml) consumed per week/1000* [27]. The average concentration of alcohol alcoholic beer in Cameroon is 5%,
5. A current smoker was defined as a participant who had smoked at least 100 cigarettes in their lifetime and still smoked at least 28 days before the interview. Participants who had smoked at least 100 cigarettes in their lifetime but had not smoked for at least 28 days prior to this study were considered ex-smoker smokers. Non-smokers were those who had smoked less than 100 cigarettes in a lifetime.

### Statistical analysis

The statistical software R (version 3.5.1, 2019, The R Foundation for statistical computing, Vienna, Austria) was used for analysis. Continuous variables were summarised using mean or median where applicable, while categorical variables were reported as frequencies and proportions. We used direct standardization techniques to compute age and sex-standardized prevalence of overweight and obesity using the 2011 population distribution of Cameroon [28]. Normality of continuous variables was assessed visually using histograms and qqplots and statistically using Shapiro-Wilk test.

Since this was a secondary analysis, we assessed the minimum detectable odds ratio that the regression analysis could detect with a power of 80%, a two-sided alpha of 0.05, and sample size of 485. For the regression analysis to identify factors associated with obesity, the minimum detectable odds ratio assuming a proportion of obesity of 0.1 in a comparison group (for example, never-smoker for smoking or male for sex) [18] was 1.9. On the other hand, the minimum detectable odds ratio for the regression analysis to identify factors associated with overweight or obesity, assuming a proportion of overweight or obesity of 0.35 in a comparison group [18], was 1.4.

Separate unconditional binary logistic regression models were used to assess factors associated with overweight or obesity (BMI > 25 kg/m^2^ versus BMI < 25 kg/m^2^), and obesity (BMI > 30 kg/m^2^ versus BMI < 30 kg/m^2^). We considered all variables with *p*-values < 0.2 on univariate logistic regression analyses qualified for inclusion in multivariable analyses [29]. Physical activity was included in the final models, as it has been shown to be associated with BMI in literature [30]. All multivariable models were adjusted for age, regardless of the level of statistical significance on univariate analyses. In all multivariable analyses, we sequentially adjusted for age and sex, sociodemographic factors (marital status, employment status, and level of education), family history of hypertension, and lifestyle factors (smoking status and physical activity).

Departure from linearity was assessed using the χ^2^ test for heterogeneity. A significant test for departures from linearity indicated that the exposure variable does not have a linear relationship with the outcome and was, therefore, modelled as a categorical variable. The χ^2^ test for linear trend was used to assess linear trend in ordinal categorical variables. Odds ratio (OR) and the corresponding 95% confidence interval (CI) was reported as the measure of the strength of the association between exposures and outcomes. The statistical software Stata 16 software (StataCorp 2019, College Station, TX: StataCorp LLC) was used to visualize results from the multivariable logistic regression analyses. The variable “Frequency of exposure to wood smoke” was excluded from the regression analysis due to more than 10% of missing data. The C-statistic was used to evaluate the predictive power of the final multiple regression model, while the Hosmer-Lemeshow test was used to evaluate the goodness-of-fit. Two-sided *p*-values less than 5% were used as the threshold for statistical significance for all hypothesis tests.

### Ethical considerations

This work is a secondary analysis. The parent study was approved by the Ethical review board of the Faculty of Health Science Bamenda and approved the West Regional Delegation of the Ministry of Public Health of Cameroon.

## Results

### Characteristics of the study population

In all, 485 (92.2%) of the initial 526 records were included in this analysis. Forty-one (7.8%) records were eliminated due to lack of data on either weight or height, which are necessary to compute the BMI.

The ages of the participants ranged from 18 to 99 years with an average of 51.9 (SD = 19.1) years. The participants were mostly females (66.0%), married (76.7%), unemployed (69.3%), and with a little over half having had less than a secondary education, Table 1. Females had a higher measure of adiposity (BMI) than males (mean BMI = 28.2 + 5.7 kg/m^2^ vs 25.4 + 4.5 kg/m^2^).

**Table 1:** Characteristics of the study population, Baham Health District, 2018

Variables^a^	Total (%), *N* = 485	Male (%), *N* = 165	Female (%), *N* = 320
Age in years^b^	51.87 (19.06)	50.23 (19.28)	52.72 (18.92)
Weight (in kg)^b^	72.17 (14.79)	73.68 (13.43)	71.38 (15.41)
Height (in m)^b^	1.63 (0.10)	1.70 (0.08)	1.59 (0.08)
Body Mass Index (in kg/m^2^)^b^	27.22 (5.44)	25.41 (4.45)	28.15 (5.68)
Body Mass Index			
Normal	189 (40.0)	89 (53.9)	100 (31.3)
Overweight	179 (36.9)	55 (33.4)	124 (38.7)
Obesity	117 (24.1)	21 (12.7)	96 (30.0)
Marital Status			
Single	113 (23.3)	51 (30.9)	62 (19.4)
Married	372 (76.7)	114 (69.1)	258 (80.6)
Employment status			
Unemployed	336 (69.3)	89 (53.9)	247 (77.2)
Employed	149 (30.7)	76 (46.1)	73 (22.8)
Occupation			
Low/Unemployed	351 (72.4)	85 (51.5)	273 (83.8)
Medium	106 (21.9)	69 (41.8)	37 (11.6)
High	21 (4.3)	11 (6.7)	10 (3.1)
Missing	7 (1.4)	2 (1.2)	5 (1.6)
Level of education			
None	122 (25.2)	30 (18.2)	92 (28.7)
Primary	139 (28.7)	39 (23.6)	100 (31.2)
Secondary	167 (34.4)	69 (41.8)	98 (30.6)
Tertiary	53 (10.9)	27 (16.4)	26 (8.1)
Missing	4 (0.8)	0 (0.0)	4 (1.2)
Family history of hypertension			
Yes	131 (27.0)	31 (18.8)	100 (31.2)
Missing	1 (0.2)	1 (0.6)	0 (0.0)
Smoking status			
Current smoker	38 (7.8)	32 (19.4)	6 (1.9)
Ex-smoker	48 (9.9)	36 (21.8)	12 (3.7)
Non-smoker	399 (82.3)	97 (58.8)	302 (94.4)
Exposure to wood smoke			
Yes	422 (87.0)	125 (75.8)	297 (92.8)
Missing	3 (0.6)	2 (1.2)	1 (0.3)
Frequency of exposure to wood smoke			
At least 4 times/week	319 (65.8)	64 (38.8)	255 (79.7)
Missing	60 (12.4)	41 (24.8)	19 (5.9)
Do you consume alcohol?			
Yes	338 (69.7)	128 (77.6)	210 (65.6)
Missing	1 (0.2)	0 (0.0)	1(0.3)
Units of alcohol consumed per week^c^	3.3 (3.3–9.8)	6.5 (3.3–16.3)	3.3 (3.3–6.5)
Duration of alcohol consumption (in years)^c^	20.0 (10.0–30.0)	20.0 (10.0–33.0)	20.0 (10.0–30.0)
Frequency of vegetable consumption (in days/week)^c^	1.0 (1.0–2.0)	1.0 (1.0–2.0)	1.0 (1.0–2.0)
Frequency of fruit consumption (in days/week)^c^	1.0 (1.0–3.0)	1.0 (1.0–3.0)	1.0 (1.0–3.0)
Intensity of daily physical activity			
Low	245 (50.5)	67 (40.6)	178 (55.6)
Medium	189 (39.0)	67 (40.6)	122 (38.1)
Vigorous	49 (10.1)	31 (18.8)	18 (5.6)
Missing	2 (0.4)	0 (0.0)	2 (0.4)

^a^All data are summarized as frequency and percentage except specified otherwise; ^b^Data summarised as mean (standard deviation); ^c^median (interquartile range) *N* Frequency

### Prevalence of overweight and obesity

The age and sex-standardized prevalence of overweight, obesity, and overweight or obesity (BMI > 25 kg/m^2^) were 31.1% (95% CI, 27.0–35.2), 18.9% (95% CI, 14.9–22.9), and 50.1% (95% CI, 45.7–54.6) respectively, Table 2. The prevalence of overweight and obesity was higher among females than males (overweight = 38.4% vs 32.7% and obesity = 30.0% vs 12.7%) and married than single participants (overweight = 39.3% vs 28.3% and obesity = 26.8% vs 15.9%), Table 2.

**Table 2:** Prevalence of adiposity by sociodemographic groups

Variables	Normal (*n* = 191) % (95% CI)	Overweight (*n* = 177) % (95% CI)	Obesity (*n* = 117) % (95% CI)
Overall crude prevalence	39.4 (35.0–43.9)	36.5 (32.2–41.0)	24.1 (20.4–28.2)
Age- and sex-standardized prevalence^b^		31.1 (27.0–35.2)	18.9 (14.9–22.9)
BMI (in kg/m^2^)^a^	22.77 (22.49–23.05)	27.51 (27.10–27.73)	33.59 (32.85–34.60)
Gender			
Male	54.5 (46.6–62.2)	32.7 (25.8–40.5)	12.7 (8.2–19.0)
Female	31.6 (26.6–37.0)	38.4 (33.1–44.0)	30.0 (25.1–35.4)
Marital Status			
Single	55.8 (46.1–65.0)	28.3 (20.4–37.7)	15.9 (10.0–24.3)
Married	33.9 (29.1–39.0)	39.3 (34.3–44.5)	26.8 (22.4–31.7)
Employment status			
Unemployed	41.4 (36.1–46.9)	36.9 (31.8–42.4)	21.7 (17.5–26.6)
Employed	34.9 (34.9–43.2)	35.6 (28.0–43.9)	29.5 (22.5–37.6)
Occupation			
Low/unemployed	39.3 (34.2–44.7)	35.9 (30.9–41.2)	24.8 (20.4–29.7)
Medium	46.2 (36.6–56.1)	34.0 (25.2–43.9)	19.8 (12.9–28.9)
High	4.8 (0.2–25.9)	57.1 (34.4–77.4)	38.1 (19.0–61.3)
Level of education			
No formal	51.6 (42.5–60.7)	32.8 (24.7–42.0)	15.6 (9.9–23.5)
Primary	36.0 (28.1–44.6)	38.8 (30.8–47.5)	25.2 (18.4–33.4)
Secondary	35.3 (28.2–43.1)	35.9 (28.8–43.8)	28.7 (22.1–36.3)
Tertiary	30.2 (18.7–44.5)	41.5 (28.4–55.8)	28.3 (17.2–42.6)

*BMI* Body mass index, *n* frequency, *CI* confidence interval, ^a^mean (95% CI), ^b^Prevalence computed using direct standardization method

### Factors associated with overweight or obesity

Table 3 shows the factors associated with overweight or obesity on univariate logistic regression analysis. There was strong evidence that females and married participants had 2.6 (OR = 2.56, 95% CI = 1.75–3.80) and 2.5 (OR = 2.52, 95% CI = 1.64–3.89) times higher odds of overweight or obesity compared to their respective counterparts. There was strong evidence of increase odds of overweight or obesity with higher levels of education. Participants who had either a secondary or tertiary education has two-fold higher odds of obesity compared to those with no formal education (OR = 2.04, 95% CI = 1.30–3.21). There was strong evidence of 74% lower odds of overweight or obesity among current smokers compared to non-smokers (OR = 0.26, 95% CI = 0.12–0.52).

**Table 3:** Factors associated with overweight and obesity on univariate logistic regression analysis

Variables	Number of cases (*N* = 296)	OR (95% CI)	*p*-value
Age below 50 years (Ref: Age > 50)	133	1.11 (0.77–1.61)	0.574*
Sex (Female)	220	2.56 (1.75–3.80)	< 0.001*
Marital status (Married)	246	2.52 (1.64–3.89)	< 0.001*
Employment (Unemployed)	199	0.7 (0.4–1.0)	0.065*
Occupation (Ref: Low/unemployed)		Ref	
Medium or high	79	1.07 (0.70–1,63)	0.763
Level of education (Ref: None)			**0.003**^$,*^
Primary	89	1.84 (1.12–3.03)	
Secondary/tertiary	146	2.04 (1.30–3.21)	
Family History of hypertension (Yes)	88	1.42 (0.39–2.19)	0.099*
Smoking status (Ref: Never smoker)	255		**< 0.001**^$,*^
Ex-smoker	29	0.86 (0.47–1.61)	
Current Smoker	12	0.26 (0.12–0.52)	
Exposure to wood smoke (Yes)	260	1.2 (0.7–2.1)	0.463
Units of alcohol consumption (Ref: Non-drinker)	83		0.585
(0.01, 6.49]	115	1.39 (0.88–2.17)	
(6.49, 117]	94	1.21 (0.76–1.92)	
Vegetable consumption (> 4 days/week)	27	1.5 (0.8–3.1)	0.267
Fruit consumption (> 4 days/week)	54	1.1 (0.8–1.6)	0.559
Intensity of daily physical activity (Ref: Low)	147		0.747*
Moderate	117	1.1 (0.7–1.6)	
Vigorous	30	1.1 (0.6–2.0)	

*Included in the multiple regression analysis; *OR* Odd’s ratio, *aOR* adjusted Odd’s ratio, *CI* confidence interval, Significant *p*-values are written in bold; ^$^*P*-value for Chi-squared test for linear trend; *Ref* Reference category

**Fig. 1:**
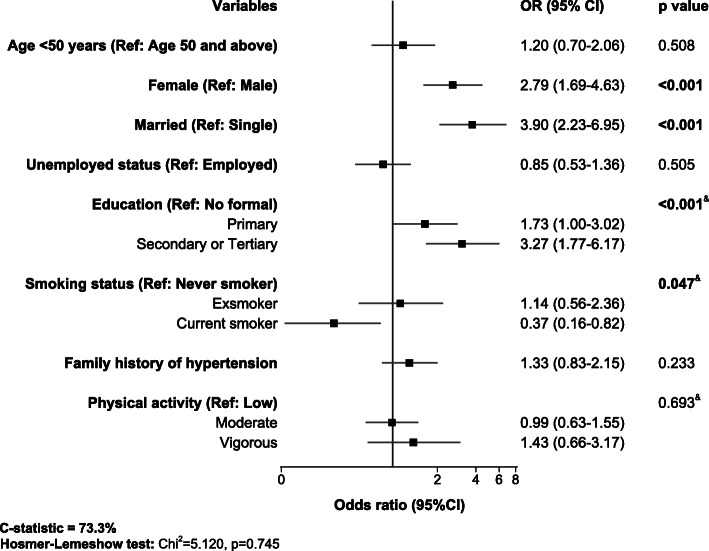
Factors associated with overweight or obesity on multiple logistic regression analysis. Measures of associations are displayed as odds ratio (OR), black squares, with the 95% confidence interval (CI), horizontal spikes. The OR and 95% CI are plotted on the logarithmic scale. Significant *p*-values are shown in bold. The solid black vertical line at OR of 1.0 refers to the null value. & = *p*-value for χ^2^ test for linear trend

The final multivariable logistic regression model was adjusted for age, sex, marital status, employment status, level of education, family history of hypertension, smoking status, and physical activity, Fig. 1. After full adjustments, there was strong evidence that female and married participants had 2.8-folds (adjusted OR [aOR] = 2.79, 95% CI = 1.69–4.63) and 3.9-folds (aOR = 3.90, 95% CI = 2.23–6.95) higher odds of overweight or obesity compared to their male and married counterparts, respectively. There was strong evidence of higher odds of overweight or obesity with increasing levels of education. Participants with primary and secondary or tertiary education had 1.7-folds (aOR = 1.73, 95% CI = 1.00–3.02) and 3.3-folds (aOR = 3.27, 95% CI = 1.77–6.17) higher odds of overweight or obesity compared to those with no formal education, respectively, Fig. 1. There was weak evidence of a 63% lower odds of overweight or obesity among current smokers compared to never-smokers (aOR = 0.37, 95% CI = 0.16–0.82). The multiple regression model had good accuracy (c-statistic = 73.3%), and the Hosmer-Lemeshow test was non-significant, indicating a good model fit (χ^2^ = 5.12, *p* = 0.745).

#### Factors associated with obesity

Table 4 shows the factors associated with obesity on univariate logistic regression analysis. Participants aged below 50 years had 1.6-folds higher odds of obesity compared to those aged 50 years or older (OR = 1.62, 95% CI = 1.06–2.47). There was strong evidence that females and married participants had 2.9 (OR = 2.94, 95% CI = 1.78–5.03) and 1.9 (OR = 1.94, 95% CI = 1.14–3.46) times higher odds of obesity compared to their respective counterparts. There was strong evidence of a higher trend of obesity with higher levels of education. Participants who had either a secondary or tertiary education had 2.2-fold higher odds of obesity compared to those with no formal education (OR = 2.94, 95% CI = 1.78–5.03).

**Table 4:** Factors associated with obesity on univariate analysis logistic regression analysis

Variables	Number of cases (*N* = 117)	OR (95% CI)	*p*-value
Age below 50 years (Ref: Age > 50)	62	1.62 (1.06–2.47)	0.025*
Sex (Female)	96	2.94 (1.78–5.03)	**< 0.001**^$,^*
Marital status (Married)	99	1.94 (1.14–3.46)	**< 0.0195**^$,^*
Employment (Unemployed)	73	0.66 (0.43–1.03)	0.065*
Occupation (Ref: Low/unemployed)	87	Ref	
Medium or high	29	0.90 (0.55–1.44)	0.660
Level of education (Ref: None)	19		**0.009**^$,*^
Primary	35	1.82 (0.99–3.03)	
Secondary/tertiary	63	2.18 (1.25–3.93)	
Family History of hypertension (Yes)	38	1.42 (0.90–2.22)	0.131*
Smoking status (Ref: Never smoker)	102		0.078^$,*^
Ex-smoker	10	0.44 (0.15–1.07)	
Current Smoker	5	0.77 (0.35–1.54)	
Exposure to wood smoke (Yes)	103	1.17 (0.62–2.33)	0.642
Units of alcohol consumption (Ref: Non-drinker)	33		0.831
(0.01, 6.49]	45	1.16 (0.69–1.94)	
(6.49, 117]	37	1.09 (0.64–1.87)	
Vegetable consumption (> 4 days/week)	10	1.10 (0.50–2.26)	0.804
Fruit consumption (> 4 days/week)	29	1.35 (0.82–2.20)	0.227
Intensity of daily physical activity (Ref: None)	58		0.991
Moderate	47	1.07 (0.68–1.66)	
Vigorous	11	0.93 (0.43–1.89)	

*Included in the multiple regression analysis; *OR* Odd’s ratio, *aOR* adjusted Odd’s ratio, *CI* confidence interval; ^$^Significant *p*-value; *Ref* Reference category

**Fig. 2:**
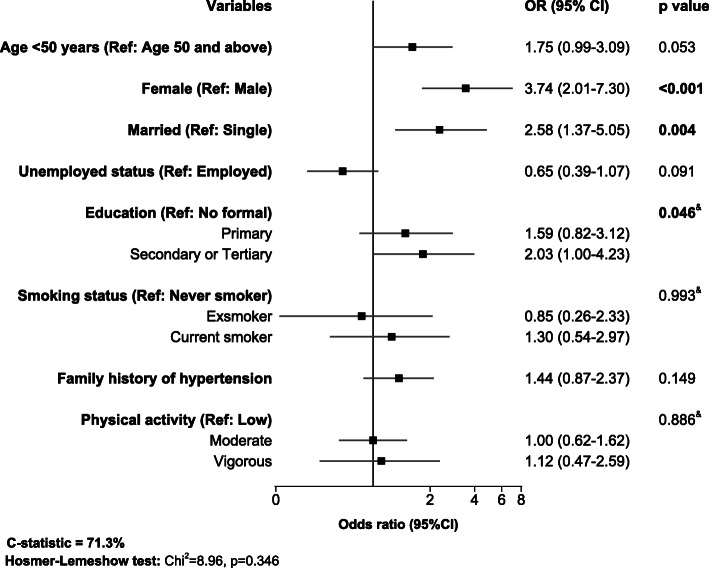
Factors associated with obesity on multiple logistic regression analysis. Measures of associations are displayed as odds ratio (OR), black squares, with the 95% confidence interval (CI), horizontal spikes. The OR and 95% CI are plotted on the logarithmic scale. Significant *p*-values are shown in bold. The solid black vertical line at OR of 1.0 refers to the null value. & = *p*-value for χ^2^ test for linear trend

We adjusted for age, sex, marital status, employment status, level of education, family history of hypertension, smoking status, and physical activity in the final multivariable logistic regression analysis, Fig. 2. After full adjustments, there was strong evidence that female and married participants had 3.7-folds (aOR = 3.76, 95% CI = 2.01–7.30) and 2.6-folds (aOR = 2.58, 95% CI = 1.37–5.05) higher odds of obesity compared to their male and married counterparts, respectively. We observed weak evidence of 1.8-folds higher odds of obesity among participants younger than 50 years (aOR = 1.75, 95% CI = 0.99–3.09) compared to those 50 years or older. In addition, there was weak evidence of higher odds of obesity with increasing levels of education and among younger participants; participants with secondary or tertiary education having two-folds higher odds of obesity compared to those with no formal education (aOR = 2.03, 95% CI = 1.00–4.23), Fig. 2. The accuracy of the multiple regression model had good accuracy (c-statistic = 71.3%), and the Hosmer-Lemeshow test was non-significant, indicating a good model fit (χ^2^ = 8.96, *p* = 0.346).

## Discussion

Adiposity is a well-known risk factor for cardiovascular disease, type 2 diabetes mellitus, and cancer [2–4]. In this rural community-based study, we found that about three in five participants were either overweight or obese. Being female, married, and having achieved higher levels of education were associated with higher odds of overweight or obesity, while current smokers had lower odds of overweight or obesity compared to their respective counterparts. On the other hand, participants aged below 50 years, female and married status participants, and those with secondary and tertiary education had higher odds of being either overweight or obese.

The age and sex-standardized prevalence of obesity of 18.9% reported in this study is about two folds that reported by Aminde et al.*,* 2017 (11.1%) in the semi-urban community of Buea, though they reported a slightly higher age-standardized prevalence of overweight of 36.5% [18]. The prevalence of obesity in our study is about six-folds higher than that reported by Sobngwi et al.*,* 2002 in rural western Cameroon [17]. The prevalence of overweight and obesity reported in this study are also higher than rates of 20.9 and 8.4% respectively reported by Adebayo et al., 2014 in a Nigerian adult rural population [31], and of 19.9 and 8.6% respectively reported recently in an Ethiopian urban setting [32]. However, the prevalence of overweight and obesity in our study are lower than those reported in previous studies in adult urban populations of Cameroon and South Africa [19, 20, 33]. Similar to Sobngwi et al, we observed an overall tendency to normal weight in our study population (mean BMI = 22.77 kg/m^2^) [17]. This is in contrast with the overall tendency to overweight observed in previous studies of semi-urban and urban populations of Cameroon [18, 34, 35]. The epidemiological transition from infectious disease to chronic non-communicable diseases in sub-Saharan Africa has been attributed most importantly to unhealthy dietary habits associated with urbanization [7, 8, 36]. A recent publication in Nature suggested that rising levels of BMI in rural areas is responsible for the global epidemic of obesity [37]. The rise in BMI in the rural areas in some developing countries was responsible for over 80% increase in global BMI. These high rates of overweight and obesity are most likely due to mechanization of agriculture, which was initially the principal source of energy expenditure in the rural areas, and increased spending on food. For instance, the development of roads, use of cars and motorbikes, provision of pipe-borne water and commercial fuel, instead of fuelwood, have drastically curb energy dissipated during agricultural activities and house chores over the years [37, 38]. Furthermore, with the social value placed on overweight, which is seen as a sign of being well-fed, and traditional energy-dense traditional meals by the people of the West Region of Cameroon [39], the mechanization of agriculture, high rates of physical inactivity, and the increasing availability of non-manual service and administrative jobs, the prevalence of overweight and obesity in this rural community could continue to rise if nothing is done.

We report that age less than 50 years, females, married status and having a tertiary education were associated with higher odds of overweight or obesity and obesity in our study population. This is in line with similar local studies [17, 18], and studies conducted elsewhere [11, 32]. The associations between a married status and obesity or overweight have been documented in previous studies conducted in the South West Region of Cameroon and other African countries [11, 18, 32, 40]. This association is likely due to the fact that, as opposed to their single counterparts, being married confers a certain degree of security and married persons are no longer concerned about attracting a partner [41]. Also, married couples tend to spend more time together, thus eat more regular and energy-dense foods [41].

Furthermore, we found that having completed higher levels of education such as secondary or tertiary education compared to no formal or primary education were associated with higher odds of being overweight or obese regardless of sex; even though one would expect learned individuals to be more informed and prone to adopting healthy lifestyles [42]. Similar findings were noted in Botswana and Tanzania [40, 43]. Individuals with higher levels of education are more likely to acquire non-manual jobs which require lesser energy expenditure compared with their counterparts with lower levels of education, who are more likely to resort to manual jobs. In addition, those with higher levels of education, who acquire skilled jobs are more likely to receive better pay checks and therefore spend more money on processed carbohydrate-containing foods despite the relatively lower physical activity, thereby aggravating the burden of overweight and obesity [11, 17, 18, 44]. However, our findings differ from some local studies and studies from South Africa, whereby having a low education was instead associated with being overweight or obese [11, 18]. Smokers had lower odds of overweight and obesity compared to never smokers. This finding was similar to that reported by Hout et al [45]. Nicotine contained in cigarette suppresses appetite, thereby preventing weight gain or even leading to weight loss [46]. Moreover, smokers are more likely to develop chronic diseases such as cancer which as associated with weight loss.

Investment in radio and television education programs about healthy eating patterns, and healthy lifestyle, promoting pre-marital education on healthy lifestyles and encouraging couples to carry out physical activities together could be relevant to reduce the prevalence of overweight and obesity in this setting.

Our study is limited by the fact that it is a secondary analysis of previously collected data. The availability and quality of the variables used in the present study were dependent on the data collected in the primary study. Our findings are subjects to both measured and unmeasured confounding. The primary study used a non-probabilistic sampling technique to recruit participating health areas and participants into the study, thereby limiting the representativeness and generalizability of our study findings. As a result, we caution against generalizing the prevalence of overweight or obesity herein. We did not assess the intraobserver and interobserver reliabilities of weight and height measurement as we only collected single measurements. However, the inter- and intra-observer reliabilities of weight and height have been reported to be excellent, especially when data collectors are trained, and electronic devices are used to measure weight [47]. Therefore, we think that measurement errors from measurements of weight and height in our study are likely to be trivial to change our findings significantly. Body mass index is a limited surrogate for adiposity. Body mass index measures excess weight rather than excess fats, and can therefore be affected by factors such as age, sex, and muscle mass [48]. Older individuals tend to have a higher BMI than younger adults which might have led to an overestimation of the prevalence of obesity and overweight in our study because our sample was constituted of relatively elderly participants. Nevertheless, BMI is a simple, available, non-invasive, and inexpensive method which is suitable to assess adiposity in epidemiological studies than more accurate and expensive methods such as dual-energy x-ray absorptiometry and bio-impedance devices [48]. Moreover, BMI is strongly correlated with adiposity and is a good predictor of morbidity and mortality [48]. A cross-sectional design does not permit us to ascertain causality. This study provides current data on the prevalence and determinants of overweight and obesity in a rural community in Cameroon. With the rising prevalence of overweight and its related complications such as hypertension, diabetes and cancer, this study provides evidence of on the factors associated with obesity and overweight in rural Cameroon.

## Conclusion

We report a high prevalence of overweight and obesity in this rural setting of Cameroon; three in five persons were either overweight or obese. Females sex, married status, and higher levels of education were independently associated with overweight and obesity in this study.

## Supplementary Information

**Additional file 1.** Map of the West Region displaying the location of the Baham Health District. (Format: pdf; Source: The map was generated by the authors using the open-source software R [version 3.5.1, 2019, The R Foundation for statistical computing, Vienna, Austria]. Shapefiles containing first level administrative data were from https://data.humdata.org/ which uses data from the open-source software CKAN [https://ckan.org/]).

## Data Availability

The dataset is available from the corresponding on reasonable request.

## Abbreviations

aOR: Adjusted Odds ratio
BHD: Baham Health District
BMI: Body Mass Index
CI: Confidence Interval
FAO: Food and Agricultural Organisation
OR: Odds Ratio
UBaMSA: University of Bamenda Medical Students Association
WHO: World Health Organisation
