# Supplementary figures and images for: Prevalence and factors associated with overweight and obesity in selected health areas in a rural health district in Cameroon: a cross-sectional analysis
Source: BMC Public Health. 2021 Mar 10;21:475. doi: 10.1186/s12889-021-10403-w (PMC7944596; doi:10.1186/s12889-021-10403-w)

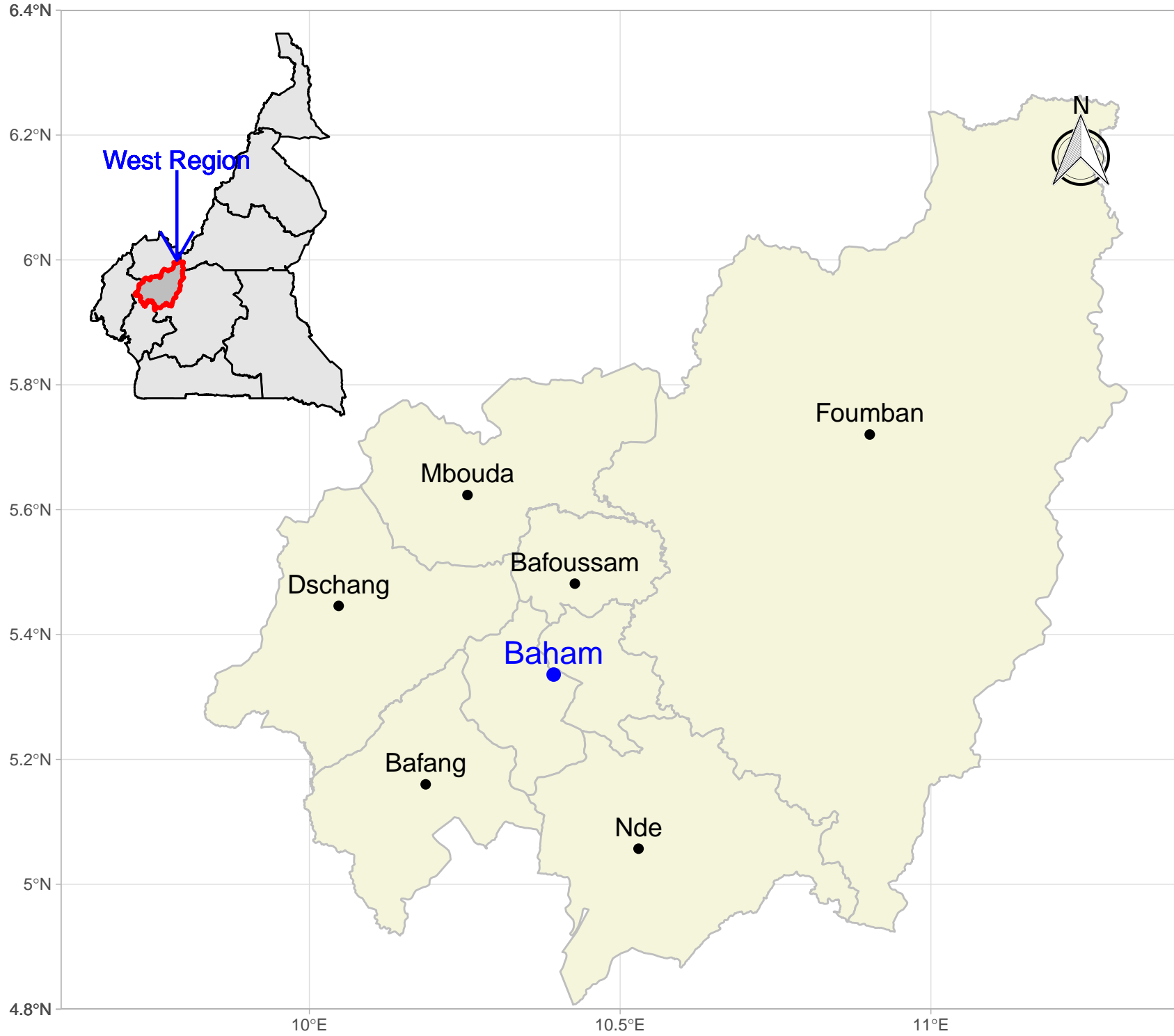

Supplement: Supplementary file 1 — Additional file 1. Map of the West Region displaying the location of the Baham Health District. (Format: pdf; Source: The map was generated by the authors using the open-source software R [version 3.5.1, 2019, The R Foundation for statistical computing, Vienna, Austria]. Shapefiles containing first level administrative data were from https://data.humdata.org/ which uses data from the open-source software CKAN [https://ckan.org/]). [file 12889_2021_10403_MOESM1_ESM.pdf]
